# Streptomycetes as Microbial Cell Factories for the Biotechnological Production of Melanin

**DOI:** 10.3390/ijms25053013

**Published:** 2024-03-05

**Authors:** Talayeh Kordjazi, Loredana Mariniello, Concetta Valeria Lucia Giosafatto, Raffaele Porta, Odile Francesca Restaino

**Affiliations:** Department of Chemical Sciences, University of Naples Federico II, via Cintia 4, 80126 Naples, Italy; talayeh.kordjazi@unina.it (T.K.); loredana.mariniello@unina.it (L.M.); giosafat@unina.it (C.V.L.G.); raffaele.porta@unina.it (R.P.)

**Keywords:** antimicrobial, antioxidant, heavy metal chelation, melanin, pigment, *Streptomyces*, UV-light protection

## Abstract

Melanins are complex, polymeric pigments with interesting properties like UV-light absorbance ability, metal ion chelation capacity, antimicrobial action, redox behaviors, and scavenging properties. Based on these characteristics, melanins might be applied in different industrial fields like food packaging, environmental bioremediation, and bioelectronic fields. The actual melanin manufacturing process is not environmentally friendly as it is based on extraction and purification from cuttlefish. Synthetic melanin is available on the market, but it is more expensive than animal-sourced pigment and it requires long chemical procedures. The biotechnological production of microbial melanin, instead, might be a valid alternative. Streptomycetes synthesize melanins as pigments and as extracellular products. In this review, the melanin biotechnological production processes by different *Streptomyces* strains have been revised according to papers in the literature. The different fermentation strategies to increase melanin production such as the optimization of growth conditions and medium composition or the use of raw sources as growth substrates are here described. Diverse downstream purification processes are also reported as well as all the different analytical methods used to characterize the melanin produced by *Streptomyces* strains before its application in different fields.

## 1. The Role and Biological Functions of Melanin

**Figure 1:**
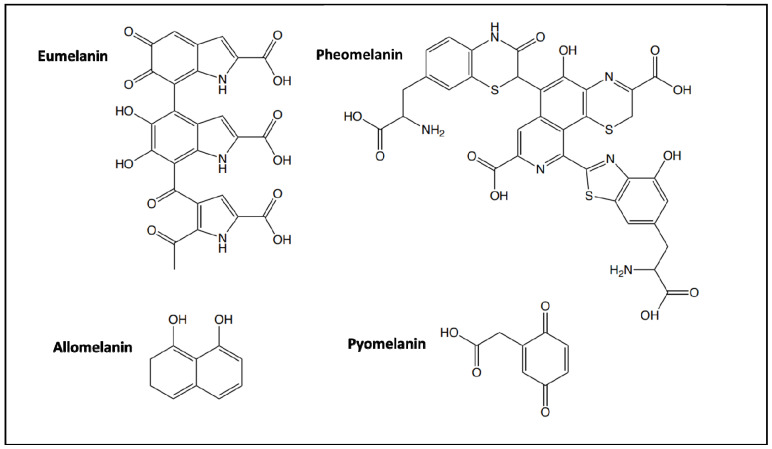
Building blocks of the polymeric structures of the different types of melanin.

Melanins are pigments that can be found in mammals, plants, and microorganisms and have the biological function of protecting against chemical and physical stresses to survive in environmentally harsh conditions like high temperatures or intensive light radiation. They are synthesized by the enzymatic-catalyzed oxidation of phenolic and/or indolic compounds that polymerize into different types of pigments like eumelanin, pheomelanin, pyomelanin, and allomelanin. Eumelanin, pyomelanin, and allomelanin have a dark black-brown color, in contrast to pheomelanin, which is red-yellow [1] (Figure 1). Mammals synthesize both eumelanin and pheomelanin. Eumelanin has a positive role by acting as a radio- and photoprotector agent and as a free radical scavenger, while pheomelanin, being less stable, might have a negative role in generating a mutagenic environment after UV exposure [2,3,4,5]. Frequently, melanin is found in the eyes and hairs of human beings and other vertebrates. In birds, instead, melanin is involved in feather coloring, which helps in signaling for reproduction [6]. In amphibians and reptiles, the dark color imparted by melanin is helpful in thermoregulation as it absorbs radiant energy [7]. In some species of cuttlefish, octopus, and squid, the production and secretion of eumelanin, in the form of ink, is a distinctive defense mechanism [8]. In mollusks, melanin is found in the shells, while in insects, instead, melanin is found in the cuticle, the outer part of the exoskeleton, and it is responsible for its sclerotization, providing protection against physical damage and pathogens [9]. In fungi, melanin assures photoprotection and resistance against chemical and mechanical stresses, while in bacteria, melanin is involved in virulence factors as well as in protection mechanisms against UV radiation and oxidative agents [10]. Some strains of *Rhizobium*, *Streptomyces*, *Marinomonas*, *Pseudomonas*, *Serratia*, and *Bacillus* species have been reported as melanin producers [11,12].

## 2. The Properties and Manufacturing of Melanin

Melanin possesses remarkable physicochemical and biological properties, such as UV–visible light photoprotection, absorption of X- and γ-rays, and antioxidant capacity against reactive oxygen and free radical species as well as redox behavior and biocompatibility [13,14]. Melanins are insoluble in many organic solvents; they show high thermal resistance and stability to chemical degradation and can be decolorized only in harsh conditions by using oxidizing agents such as KMnO_4_, NaOCl, and H_2_O_2_ [1]. Melanin pigments are also amorphous semiconductors, and they can act as heavy metal chelators. Thanks to all these properties, and being polymeric molecules, melanins have been used in different application fields, for example, as antioxidants in drug formulations, as sunscreen or hair dyes in cosmetics, as pigments for lenses of sunglasses in the optical industry, as heavy metal chelators in the environmental bioremediation processes of soil and water, as components of electronic circuits, batteries, and solar cells, or as additives in synthetic polymers or nanoparticles and also in 3D scaffolds for biomedical applications [15,16,17,18,19]. Nowadays, the manufacturing process of melanin is based on extraction from animal sources like the ink of cuttlefishes such as *Sepia officinalis*. The extraction is not an environmentally friendly procedure as it depends on the availability of animal sources, which must be sacrificed in high numbers to obtain a substantial amount of the pigment, and it requires long purification processes to remove residual proteins and nucleic acids contemporarily withdrawn from the tissues. All this increases the pigment’s final cost (1 g of melanin from *Sepia officinalis* costs almost EUR 470, according to the SigmaAldrich website [20,21]). Extracting the pigment from plants is even more expensive as the concentration of melanin in plant tissues is very low. Synthetic melanin is also available on the market, but its production requires long chemical procedures, and the final pigment structure does not always resemble a natural-like pigment structure. The latter manufacturing process is even more expensive than the animal one (1 g of synthetic melanin costs almost EUR 775, according to the SigmaAldrich website, [20]). The biotechnological production of microbial melanin, instead, might be a valid alternative as fungi and bacteria might synthesize it as a pigment. Fungi can produce melanin as an intracellular product at a g/L scale but in very long processes that last at least 14 days [22]. Among bacteria, some *Streptomyces* strains might produce melanin as a secondary metabolite in a shorter time, in 5–6 days, at a g/L scale as well, but as an extracellular product that could be more easily recovered. However, the optimization of melanin production by *Streptomyces* strains, and the modification of specific biosynthesis pathways to overproduce it, has been rarely investigated so far [23]. To set up a biotechnological process of melanin production by *Streptomyces*, an informed strain selection must be performed as well as the optimization of growth conditions, like pH and temperature, or of the medium composition also in terms of metal ion or amino acid content, etc. The application of genetic engineering techniques to enhance the activity of the enzymes involved in melanin formation, such as tyrosinase, could also lead to improved productivity. In the next paragraphs, the different approaches reported in the literature for the biotechnological production of melanin by diverse *Streptomyces* strains will be highlighted as well as the various aspects of the pigment purification and structural characterization, and further research approaches and innovative strategies will be suggested.

## 3. *Streptomyces* Strains as Melanin Producers

**Figure 2:**
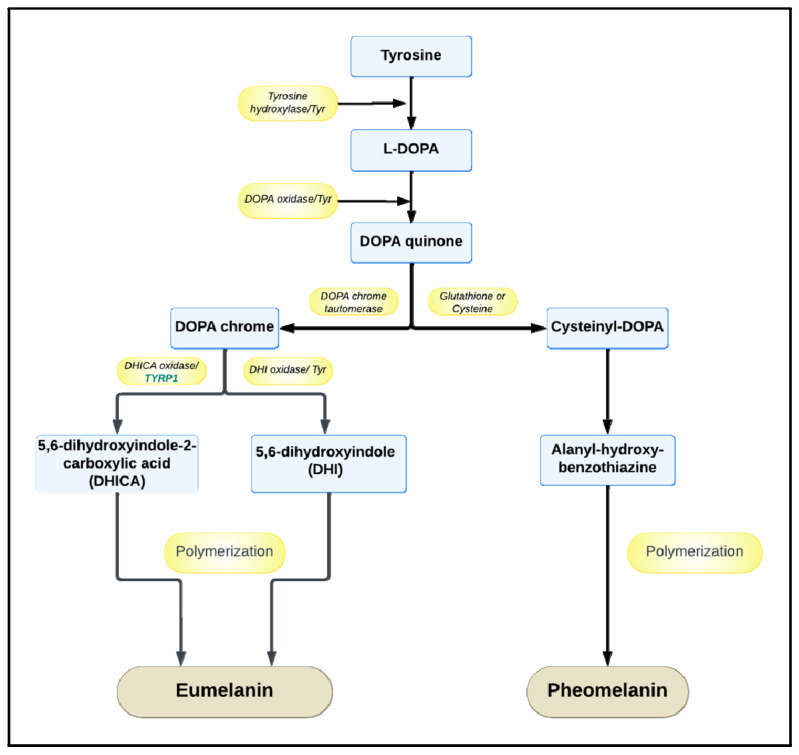
Metabolic pathways for the biosynthesis of the different types of melanin in *Streptomyces* strains.

**Figure 3:**
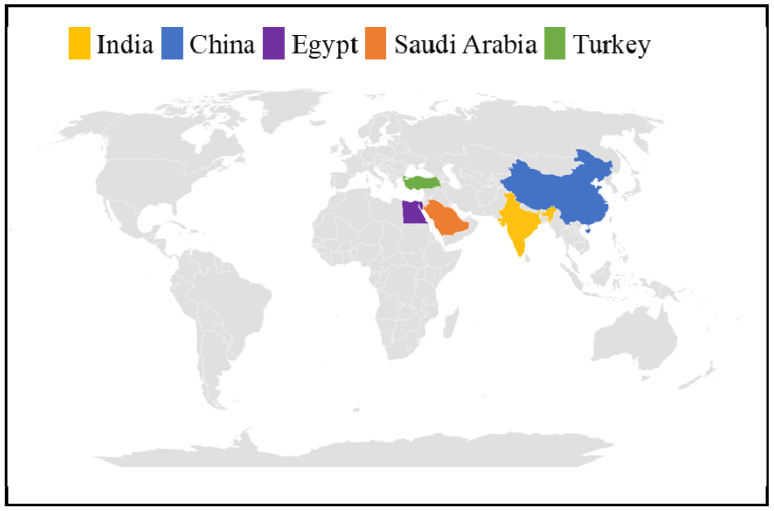
World map showing the countries where the new *Streptomyces* strains able to produce melanin have been more frequently isolated, according to the literature data.

*Streptomyces* strains mainly produce dark brown eumelanin (or DOPA-melanin) or yellow-red pheomelanin-like pigments, according to precise melanin biosynthetic pathways that involve, as the first enzyme, tyrosinase, a mono-oxygenase having a di-nuclear copper catalytic center able to catalyze both the ortho-hydroxylation of monophenols (cresolase activity) and the oxidation of catechols (catecholase activity), generating ortho-quinone products [24] (Figure 2). Tyrosinase first catalyzes the hydroxylation of L-tyrosine to 3,4-dihydroxy-L-phenylalanine (L-DOPA) by employing the molecular oxygen bound to the catalytic center. Then, in the case of eumelanin, L-DOPA is oxidated into DOPA-quinone and then converted further into DOPA-chrome thanks to a DOPA-chrome tautomerase. The DOPA-chrome is then further oxidated into 5,6-dihydroxyindole-2-carboxylic acid (DHICA) and 5,6-dihydroxyindole (DHI) precursors that polymerize to form eumelanin. In the presence of glutathione or cysteine, instead, L-DOPA is converted into an alanyl-hydroxy-benzothiazine precursor that then polymerizes into pheomelanin [25] (Figure 2). The initiation of melanin synthesis in *Streptomyces* depends on two genes belonging to an operon, i.e., *melC1* and *melC2*, that also regulate the activation of an apotyrosinase form and its subsequent secretion in the external medium as tyrosinase enzyme [26,27]. The MelC1 protein determines the effective secretion of tyrosinase in the medium, but mutants, defective in the *melC1* gene and thus in the secretion of the enzyme, have also been found [27]. In the literature, several new *Streptomyces* strains that can produce melanin were isolated from soil samples [22,28,29,30,31,32,33,34,35] from different locations in Egypt, India, and Turkey, or from forest or lake environments, also eventually associated with rhizosphere or plants [32,36,37,38,39] (Figure 3). Some others, instead, have been isolated from marine sediments [40,41,42,43,44,45] in India and China, for example, or eventually associated with marine animals like the sea cucumber *Stichopus vastus* [46] (Figure 3). According to the strain, differences among the melanin pigments were noted in terms of color first, and then in terms of the structure and/or properties. For example, two isolated strains, including *Streptomyces* sp. RF-2 and *Streptomyces* sp. BJZ10, both produced brown melanin [28], while a soluble darker pigment was produced by three marine *Streptomyces* strains named F1, F2, and F3 [42]. Melanin pigments with a diverse range of colors from greenish-brown to brownish-black were produced at the same time by an isolated marine actinomycete [41]. The pigment produced by *Streptomyces parvus* BSB49 had a eumelanin-like structure [30], and a pheomelanin-like pigment was produced by a marine *Streptomyces* sp. [43], while *Streptomyces djakartensis* NSS-3 was one of the very rare strains reported so far to be able to produce pyomelanin (El-Zawawy et al., 2024). *Streptomyces cavourensis* RD8, a marine actinomycete, produced melanin with very high antioxidant activity [40]. *Streptomyces lusitanus* DMZ-3 was able to synthesize both insoluble and soluble melanins [31], while *Streptomyces bellus* MSA1, isolated from marine sediments, also demonstrated the production of high contents of the pigment as an intracellular product [29]. In other papers, strains belonging to microbial cell banks, not newly isolated ones, like *Streptomyces roseochromogenes* ATCC 13400 and *Streptomyces nashvillensis* DSM 40314, were reported as good melanin producers as well [23,47]. Engineered *Streptomyces* strains have also been built to overproduce melanin by overexpressing the genes encoding tyrosinase and by inserting them in a replicative plasmid vector under the control of an inducible promoter [31]. In the case of *Streptomyces kathirae* SC-1, the tyrosinase enzyme was first purified and then characterized, showing a molecular weight of 30 kDa, with a Km for L-tyrosine and L-DOPA of 0.25 and 0.42 mM, respectively [48]. Then, the amino acid sequence was determined and employed to design primers that allowed for the amplification of the *melC1* gene, which is responsible for tyrosinase expression, and of its region containing two putative promoters called Pskmel and P135. The gene *melC1* was cloned under the transcriptional control of either the putative promoter or the constitutive promoter PermE in the replicative plasmid pIJ86, and the resulting constructs were inserted in both *S. lividans* and *S. kathirae* cells [48]. Tyrosinase genes from *Streptomyces lusitanus* and *Streptomyces kathirae* were also successfully expressed in *Escherichia coli*, and the recombinant strains were able to produce eumelanin when grown on L-tyrosine-supplemented medium [38]. In another example, instead, the tyrosinase genes from *Streptomyces antibioticus* were inserted in another recombinant *E. coli* JM109 strain and put under the transcriptional control of a phage T5 promoter and of two lac operators [39].

## 4. Biotechnological Strategies to Improve Melanin Production by *Streptomyces* Strains

Physiological factors, including environmental conditions, play an important role in the enzymatic activity and secondary metabolism of *Streptomyces* strains [49,50]. Optimization studies of parameters, like pH, temperature, carbon and nitrogen sources, the salinity of the medium, inoculum size, time of growth (2–30 days), agitation speed (100–250 rpm), and the ratio between the medium volume and total air volume, have been performed in the literature to increase the melanin production [22,23,28,29,30,31,40,51,52,53]. Although pH and temperature conditions are key factors for secondary metabolite synthesis in Streptomycetes [50], only very few papers have performed a systematic study of the best pH and temperature values for each strain, as in the case of *Streptomyces cavourensis* RD8 [40], *Streptomyces nashvillensis* DSM 40314 [47], and *Streptomyces* sp. ZL-24 [32], for which the best growth conditions resulted to be pH 6.0 and 30 °C, pH 7.0 and 28 °C, and pH 7.0 and 30 °C, respectively. In other papers, pH values between 6.0 and 8.5 and temperatures in the range between 26 °C and 50 °C have been employed to produce melanin without performing any initial physiological optimization studies (Table 1). For example, *Streptomyces kathirae* SC-1 was directly grown at pH 6.0 and 28 °C [31], while *Streptomyces roseochromogenes* ATCC 13400 was cultivated at pH 6.0 and 26 °C [23] (Table 1). In the case of *Streptomyces* sp. ZL-24 [32], the nutrient composition of the medium was also studied in initial physiological experiments to improve melanin production. In fact, it was demonstrated that the production of microbial melanin is greatly affected by the medium components like diverse carbon and nitrogen sources as well as by salts [22,29,30] (Table 1). The main reported carbon sources were glycerol, glucose, galactose, fructose, xylose, arabinose, rhamnose, raffinose, mannitol, inositol, sucrose, lactose, melibiose, starch, dextrose, and maltose [22,23,29,30,31,32,33,36,40,44,46,52], while both simple nitrogen sources, like ammonium sulfate, ferric ammonium citrate, sodium or potassium nitrate, ammonium chloride, ammonium nitrate, and urea, and complex nitrogen sources, like yeast extract, soybean meal, soy peptone, malt extract, beef extract, casein, casein peptone, meat peptone, and protease peptone, were exploited [22,23,29,31,32,34,40] (Table 1). Many amino acids were included in different media like glycine, cystine, alanine, tryptophan, valine, leucine, proline, glutamine, aspartate, asparagine, phenyl alanine, and histidine, and L-tyrosine was often added to the medium as the first precursor of the biosynthetic pathway to enhance melanin production [31,34,35,40,44,52]. Salts like MgSO_4_, NaCl, FeSO_4_, K_2_HPO_4_, Na_2_HPO_4_, CaCO_3_, KCl, Na_2_S_2_O_3_, KH_2_PO_4_, CaCl_2_, NiCl_2_, FeCl_2_, MgCl_2_, ZnCl_2_, MnCl_2_, CaCl_2_, and CoCl_2_ were tested as medium components, which affected the melanin production as well [29,31,44] (Table 1). Frequently, optimization experiments were performed by employing experimental design models like the one-factor-at-a-time (OFAT) method, Response Surface Methodology as Plackett–Burman design (P-BD), Central Composite Design (CCD), and face-centered central composite design [22,34,35,40]. Experiments were performed both on solid agar media and in liquid; in the last case, they were mainly performed in shake flasks except for only one case in which batch fermentations were run in a 2.5 L fermenter [23] (Table 1). By using these different approaches, melanin concentrations in the papers varied in the range of 0.087 to 13.7 g/L (Table 1). For example, the marine strain *Streptomyces* MVCS13 was able to produce extracellular melanin at different concentrations (from 0.023 to 0.147 g/L) according to the pH, temperature, and medium components [44] (Table 1). A surface response method was employed to optimize the medium and growth conditions of *Streptomyces kathirae* SC-1, which displayed the highest capacity for melanin production among all the isolates, up to 13.7 g/L [31] (Table 1). Assays of the secreted tyrosinase were performed in several cases to correlate the enzymatic activity with the quantity of melanin produced in different conditions, for example, in the cases of *Streptomyces* sp. ZL-24 [32], *Streptomyces roseochromogenes* ATCC 13400 [23], and *Streptomyces nashvillensis* DSM 40314 [47]. The determination of tyrosinase activity might also be useful in correlating the enhancement in melanin production with supplementation to the growth medium of some metal ions that might influence both the gene transcription of tyrosinase synthesis as well as its activation from an apo-tyrosinase form [26,27,53,54,55]. For example, ferrous (III) and nickel (II) ion additions to the growth medium had a boosting effect on the melanin production of *Streptomyces* sp. ZL-24 [32]. The optimal medium formulated with 0.39 g/L of NiCl_2_, 1.33 g/L of FeSO_4_, and 20.31 g/L of soy peptone resulted in the maximum production of insoluble melanin of 0.189 g/L, which was obtained in 5 days of growth at pH 7.0 and 30 °C, starting with 3% (*v*/*v*) of inoculum. A soluble version of the pigment was instead produced up to a concentration of 4.24 g/L when the strain was grown on solid agar plates of the same optimized medium [32] (Table 1). In fact, in many cases in the literature, growth on solid agar media was employed to produce melanin. For example, *Streptomyces* sp. EF1 produced a dark brown pigment when cultured on nutrient agar plates supplemented with 0.4% L-tyrosine and L-DOPA [56], while *Streptomyces puniceus* RHPR9 synthesized a substantial amount of extracellular melanin pigment, up to 0.386 g/L, on agar medium containing peptone, yeast extract, and iron [36]. The cost-efficient production of melanin at a large scale is technically challenging. Alternative production routes based on the use of raw lignocellulose sources as substrates for microbial growth and melanin production have been explored in the last few years. The strategy of valorizing low-cost and abundant agro-industrial wastes complies with the concept of sustainable development and a circular bioeconomy. Streptomycetes can degrade lignocellulose biomasses thanks to their multi-enzymatic pools that include different cellulases (endo-, exo-, and betagalactosidases), lignin peroxidases, and/or laccases. For example, recently, egagropili sea balls, derived by the aggregation of roots and rhizomes of the marine seagrass plant *Posidonia oceanica* that accumulated as wastes along Mediterranean Sea coasts [57], were explored as raw sources to enhance the extracellular melanin production of *Streptomyces roseochromogenes* ATCC 13400 [23]. Different concentrations (1.0, 2.5, and 5.0 g/L) of untreated egagropili powder were supplemented into a culture medium containing glucose, yeast, and malt extracts. The melanin production was enhanced in shake flasks by 7.4-fold, compared to the control, resulting in up to 3.94 ± 0.12 g/L and up to 9.20 ± 0.12 g/L in 2 L batches, in 96 h runs, with a productivity of 0.098 g/L/h (Table 1). The boosted melanin production was due to the synergistic effect of both the cellulose components of the egagropili, which supported the growth, and of the lignin fraction that enhanced the pigment production [23]. Another recent study showed the effect of distinctive concentrations of fava bean seed peel supplementation, as a natural source of L-tyrosine, on the melanin production of *Streptomyces cyaneus*. Different concentrations of peels were added to an optimized medium (0.5, 1.0, 1.5, 2.0, and 3.0%). The best concentration of peels (2.0%) increased the pigment production up to 9.9 g/L and the tyrosinase activity up to 497.0 U/mL [33]. Then, by irradiating the culture with gamma rays, the melanin synthesis was further increased up to 1.1 g/L (Table 1). To optimize the melanin production by *S. antibioticus* NRRL B-107, instead, a hydrolysate residue of *A. platensis* was used as a low-cost source of L-tyrosine. The concentration of various medium components, including yeast extract, soluble starch, CuSO_4_, and the *A. platensis* hydrolysate itself, was optimized through a CCD model. The results indicated a significant effect of yeast extract and the hydrolysate supplementation on melanin production, up to a maximum of 0.24 g/L [39] (Table 1). *Streptomyces antibioticus* NRRL B-1701 also showed an ability to use non-natural amino acids, such as N-acetyl-L-tyrosine and L-tyrosine ethyl ester, as substrates for tyrosinase activity [39]. Higher melanin concentrations were reached only with recombinant strains like *S. kathirae*, which had multiple copies of the *melC* gene, and it was able to produce up to 28.8 g/L of the pigment (Guo et al., 2015). The expression of this gene in the recombinant *E. coli* JM109 strain resulted in reaching only 0.4 g/L of eumelanin on the LB medium (Table 1).

## 5. Purification Processes for the Melanin Produced by *Streptomyces* Strains

Purification processes might significantly impact the biotechnological production of melanin in terms of the overall costs, timing of the process, number of cycles that must be repeated, scalability, pureness, and recovery percentages of the pigment [43,58,59]. *Streptomyces* strains can produce melanin as an extracellular product. Thus, its recovery from the clarified broth supernatant, as the first step of the purification process, is easier than the alkali-based extraction that is usually performed to recover the intracellular pigment from fungi cells [43,58,59]. Although the isolation and purification of melanin require the use of different steps, diverse methods have been tested so far, like precipitation, extraction, dialysis, and chromatography [58]. A summary of these methods is reported in Table 2. One of the difficulties in designing and setting up a purification protocol for melanin is the low solubility of the pigment in many organic solvents and acidic aqueous solutions at a pH lower than 2.0 [43,59]. Knowing the solubility of the produced melanin could be an advantage in establishing the initial isolation step. In the first stages, after the pigment is secreted into the culture broth, centrifugation is commonly employed to remove the biomass and recover the clarified broth supernatant. To partially separate the pigment from other metabolites and from the exhausted medium components present in the supernatant, acidic precipitation and liquid–liquid solvent extractions have been employed (Table 2). Adjusting the pH to acidic conditions (at pH values lower than 2.0) might be a wise strategy to induce the precipitation of pigment. Frequently, instead, testing the solubility of the pigment in solvents with diverse polarities, like methanol, ethanol, hexane, etc., is useful to then recover the melanin from the supernatant by using a liquid–liquid extraction. In some studies, physical methods, like sonication, were also employed to extract the remaining intracellular pigment (Table 2). The following steps usually included membrane filtration, dialysis, or chromatography purification. The repetition of specific steps in the purification process might vary, and multiple centrifugations, extraction cycles, repeated crystallizations, and lyophilization procedures have been frequently reported [58,59] (Table 2).

## 6. Analytical Methods for Melanin Characterization

The chemical structure of melanin is highly complex and its polymeric nature, being made of different indolic or phenolic units, makes its determination a critical issue [58]. *Streptomyces* strains mainly produce eumelanin-like (or DOPA-melanin) or pheomelanin pigments that have different colors, structures, and chemical compositions. In the literature, several analytical methods have been reported to determine the structure of the pigments produced and purified by the diverse *Streptomyces* strains like ultraviolet–visible (UV–visible) and Fourier-transform infrared spectroscopy (FT-IR), nuclear magnetic resonance (NMR) and elemental analysis, and scanning electron microscopy (SEM). All these techniques give complementary information that is useful in characterizing the type of melanin. Here, an overview of the different analytical approaches used in the literature and of the structural data of the diverse melanins produced by Streptomycetes are summarized.

### 6.1. Ultraviolet–Visible Spectroscopy

**Figure 4:**
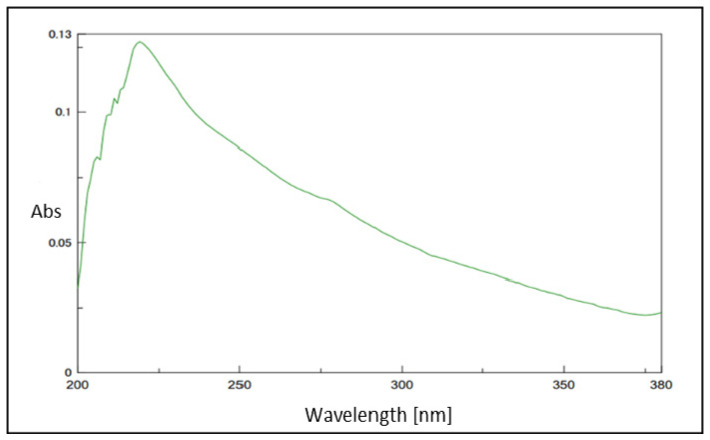
A representative UV spectrum of melanin produced by *Streptomyces* strains.

Ultraviolet–visible (UV–visible) spectroscopy is a widely used technique to study absorption in the ultraviolet and visible regions of a pigment whose spectrum has peculiar features. Regarding the melanin produced by animals or by other microbial species, the pigments produced by *Streptomyces* strains show an absorption profile over the entire UV range with a typical monotonic decay curve that gradually diminishes in the visible spectrum. One or more maximum peaks of absorbance in the range between 200 and 350 nm might be noted (Figure 4). The maximum peaks might change according to the strain and the intricate conjugated structures of phenolic and/or indolic groups [22,29,30]. Analyses are frequently performed in alkali solutions (i.e., 0.1 M NaOH solution) in which melanin pigments are generally soluble. For example, the melanin produced by *Streptomyces* BJZ10 showed a maximum at 247 nm [28], while the melanins produced by *Streptomyces glaucescens* NEAE-H [22], *Streptomyces parvus* BSB49 [30], and *Streptomyces puniceus* RHPR9 [36] revealed a peak of maximum absorption at 250 nm (Table 3). In some cases, the strains produced two different pigments, i.e., soluble and insoluble ones, that can show differences in absorption, as in the case of the melanins produced by *Streptomyces* sp. ZL-24, which exhibited two peaks at 207 nm and 213 nm [32]. Insoluble and soluble melanins produced by *Streptomyces lusitanus* DMZ-3, instead, exhibited the same UV absorption profile in the range between 200 and 300 nm, with a maximum at 230 nm [52] (Table 3). The melanin generated by the engineered strain *Streptomyces kathirae* SC-1 showed a peak at 220 nm and an absorption profile similar the one of commercial melanin standards [31] (Table 3). Sometimes, UV–visible analyses were performed after some purification steps, for example, after a reverse-phase chromatography procedure employed to purify the melanin produced by *S. roseochromogenes*. In this case, the pigment showed two sharp peaks at 222 nm and 254 nm and a smaller peak at 332 nm [23]. Also, the purified melanin produced by *Streptomyces* sp. MVCS13 showed an absorption peak at 300 nm [44]. The precipitated and then purified melanin of *Streptomyces cavourensis* SV 21 showed, instead, an intensified absorption peak at 260 nm [46]. Together with the determination of the maximum peaks in the UV wavelength range, another criterion to identify melanin consists of plotting the logarithm of the absorbance in the visible range against the wavelengths (420–600 nm) [43,60]. If the plot reveals a linear relationship with negative slopes, it certifies the melanin structure, as reported in the case of the pigment produced by *Streptomyces* sp., which showed a specific slope value of −0.0028 [43].

### 6.2. Fourier-Transform Infrared Spectroscopy

**Figure 5:**
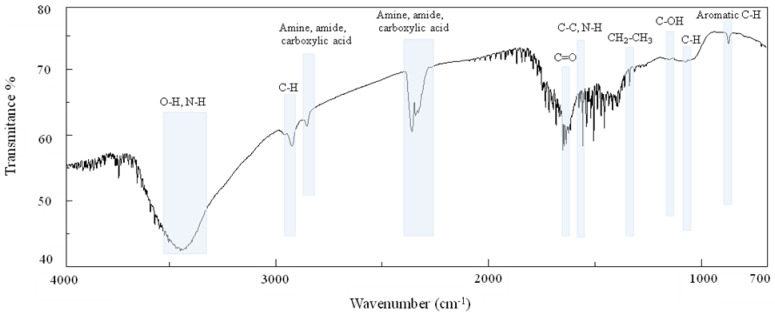
A representative FT-IR spectrum of melanin produced by *Streptomyces* strains.

Fourier-transform infrared spectroscopy (FT-IR) is a technique frequently used to characterize the structure of melanin from different sources as the pigment shows some characteristic, distinctive bands typical of the major functional groups (Figure 5) [61]. In general, purified melanin pigment is ground with KBr powder in an agate mortar (in a ratio of 1 or 2 mg of melanin to 200 mg of KBr), mixed, and set up in the form of a disc that is then scanned between 4000 and 400 cm^−1^. The different origins of melanin, its solubility, and the procedures employed to purify it, slightly change the FT-IR signals [39] (Table 4). A summary of the main FT-IR signals of the different types of melanin produced by the diverse *Streptomyces* strains, as found in the literature, is reported in Table 4. Indicatively, the FT-IR spectra of the pigments show the first broad peaks within 3400–3200 cm^−1^, due to the stretching vibrations of the -OH and -NH groups of the indolic and pyrrolic rings, and frequently small signals between 2964 and 2850 cm^−1^, due to the stretching vibration of aliphatic C-H groups (Table 4). Broad signals around 2300 and 2800 cm^−1^ have frequently been attributed to the stretching vibrations of the O-H and N-H bonds of amine, amide, or carboxylic acid present in the indolic and pyrrolic rings (Table 4). Signals of the C=O stretching of quinone or carboxylic acid groups can be found as multiple signals around 1700 cm^−1^, while signals between 1661 and 1523 cm^−1^ are considered to be due to the stretching of the aromatic C=C groups and to the bending of secondary N-H bonds (Table 4). Additional bands between 1532 and 1515 cm^−1^, also due to N–H bending, usually indicate the presence of an indole structure in the polymer. Multiple signals at 1450–1400 cm^−1^, characteristic of the melanin pigment, depend on CH_2_-CH_3_ bending, while signals in the region of 1305–1250 cm^−1^, instead, are characteristic of aromatic esters. Small peaks around 1260 and 1150 cm^−1^ are usually due to the stretching of phenolic C-OH groups (Table 4), while bands between 1112 and 1129 cm^−1^ are associated with C-N stretch related to aliphatic amines. Other characteristic signals of melanin are the ones between 1050–1095 and 880–800 cm^−1^, which are due to the bending of in-plane aliphatic and aromatic C-H groups, respectively (Table 4), while the eventual presence of weak bands below 700 cm^−1^ generally represents C-H alkene groups. In the case of the presence of sulfur atoms in the structure, as in the pheomelanin-like pigments, small signals of S=O and C-S stretching can also be found at 1420–1380 and 662 cm^−1^, respectively. Thus, these signals might be useful in differentiating the type of produced melanin. In many papers, FT-IR analyses were used just to confirm that the produced pigment was melanin, but frequently, the authors did not determine the exact structure. Therefore, multi-analytical approaches, including NMR or elemental analyses, are necessary (Table 4).

### 6.3. Nuclear Magnetic Resonance Spectroscopy

Sometimes, to confirm the molecular structure of the melanin produced by *Streptomyces* strains, further analyses have been performed by using mono- (^1^H and/or ^13^C) and bi-dimensional (COSY and/or HSQC) nuclear magnetic resonance spectroscopy (NMR) [40]. In the literature, this technique is not frequently employed to characterize biotechnologically produced melanin because the low solubility of the pigment makes it difficult to find the right solvent to dissolve it (NaOH and DMSO are the most used solvents used to perform NMR analyses of melanin). In addition, this analytical technique also requires a highly pure sample and more effort to eventually remove residual proteins that easily form weakly bonds with melanin. Despite this, NMR analyses could provide great information on the polymeric structure of the pigment as well as on its pureness. In ^1^H NMR spectra, broadening signals in the region ranging from 6.0 to 8.0 ppm were generally attributed to conjugated aromatic moieties and, specifically, resonances observed at 7.60, 7.35, 7.00, and 6.60 ppm were usually considered characteristics of the indole and/or pyrrole repeating units of melanin as a result of its irregular nature of polymer [61]. For example, the signals of melanin produced by *Streptomyces parvus* BSB49 clearly corresponded to the aromatic groups and, specifically, to the indole/pyrrole repeating units of the pigment structure [30]. Signals between 3.20 and 4.30 ppm, instead, were considered to be due to the protons bound to the N or O atoms, while the resonances falling within the 1.00 to 3.20 ppm range were assigned to the aliphatic region [32]. The ^1^H NMR spectrum of the purified melanin pigment produced by *Streptomyces glaucescens* NEAE-H [22] revealed the distinctive resonances of the aromatic functionalities at chemical shifts ranging between 8.0 and 6.0 ppm, as well as signals spanning from 3.2 to approximately 4.3 ppm of the protons associated with nitrogen and/or oxygen atoms. These assignments were similar to the signals found for both human hair and *Sepia officinalis* melanin. Melanin obtained from *S. puniceus* RHPR9 displayed signals in the range of 8.3 to 6.0 in the ^1^H NMR spectrum, which were again associated with indole or pyrrole repeating units, while peaks in the range of 0.973 to 4.282 ppm were attributed to protons linked to methyl or methylene groups connected to nitrogen and/or oxygen atoms. Instead, signals between 1.0 and 3.00 ppm were considered indicative of the presence of an indole-linked NH group, and a peak at 0.9 ppm corresponded to an aliphatic methyl group [36]. The ^1^H NMR spectrum of *Streptomyces* sp. ZL-24 [32] melanin also exhibited strong resonances in the range of 4.5 to 5.2 ppm, which were identified as anomeric protons associated with C=C groups, together with signals in the range of 3.2 to 4.3 ppm. Sometimes, inaccuracies might occur in the assignments of the signals in the region between 2.50 ppm and 3.33 ppm, as the presence of acidic OH/NH signals might either overlap with the residual solvent peaks or remain undetected due to rapid deuterium–proton exchange in DMSO-d6 [30]. Resonance signals that appeared in the field of the spectrum between 0.50 and 2.30 ppm, instead, were usually considered indicative of residual protein peaks [22,30]. An accurate purification procedure before performing both mono- and bi-dimensional NMR analyses could help to overcome this issue and to better correlate proton signals with carbon signals. For example, the ^1^H and COSY NMR spectra of the melanin produced by *Streptomyces roseochromogenes*, purified by reverse phase chromatography, exhibited various signals in both the aromatic and up-field regions that correlated with each other and were useful in determining the eumelanin-like structure of the pigment [23]. In the aromatic region, two proton signals at δ 6.53 and 6.95 ppm correlated with the carbon signals at δ 118.9 and 129.4 ppm of the two C2 and C3 positions of the 5,6-dihydroxyindole units, respectively. More shielded protons at δ 7.30 ppm, instead, were correlated with carbon at δ 128.6 in an HSQC experiment; these signals were attributed to the H3/C3 of the 5,6-dihydroxyindole-2-carboxylic acid units. The presence of carboxyl functional groups was confirmed by characteristic signals in the ^13^C spectrum [23]. A simpler downstream procedure, consisting of two steps of precipitation and washing with acidic solutions, was used to obtain a purified form of the melanin produced by *Streptomyces nashvillensis* DSM 40314. The melanin was found to have a purity of 75%, and the protein fraction associated with the pigment was almost completely removed, as evident from the low number of protons signals in the 2.6–4 ppm region of the ^1^H NMR spectrum [47]. By analyzing the purified pigment also by ^1^H,^1^H COSY, the signals of the catechol ring system at 6.64, 7.01, and 7.22 ppm were also found to be correlated with each other, while the signals at 7.17, 7.30, and 7.41 ppm of the 5,6-dihydroxyindole ring system were also clearly visible [47].

### 6.4. Elemental Analysis

Diverse methodologies have been applied for melanin elemental analysis, like energy dispersive X-ray and elemental combustion analysis. These techniques provide insights into the elemental composition of the different types of melanin based on the carbon, hydrogen, oxygen, nitrogen, and sulfur percentages and help to determine their structures. For example, very low percentages (i.e., 0.09%, lower than 1.0%) of sulfur atoms are indicative of a eumelanin-like pigment, while higher values (from 2.0% to 10.0%) are indicative of pheomelanin structures. Sulfur-containing melanins typically consist of benzothiazine or benzothiazole compounds [25,62]. Elemental analysis could also be useful to discriminate the eumelanin and pheomelanin pigments produced by *Streptomyces* strains from the conventional allomelanins mainly produced by fungi, which lack nitrogen content [63]. For example, the extracellular melanin produced by marine *Streptomyces* sp. showed an elemental composition of 45.79% carbon (C), 3.89% hydrogen (H), 9.12% nitrogen (N), 39.08% oxygen (O), and 2.12% sulfur (S), suggesting a pheomelanin type pigment as the predominant component [43]. The melanin produced by *S. hyderabadensis* 7VPT5-5R, instead, showed an elemental analysis composition of 27.38% carbon (C), 4.50% hydrogen (H), 5.08% nitrogen (N), and 0.88% sulfur (S), suggesting a eumelanin-like structure for the pigment [37]. Purification procedures seemed to not significantly change the melanin composition as verified by comparing the elemental composition of the crude and the purified pigment by *Streptomyces cavourensis* RD8, which showed similar C (46.76 and 36.59%) and S contents (0.84 and 0.62%), respectively [40]. The elemental molar ratios of C:H, O:C, and C:N could also provide insights into the structure of the oligomer units as a low C:N molar ratio suggests the presence of amino groups, while a low C:H molar ratio indicates a higher proportion of heterocyclic structures [64,65].

### 6.5. Scanning Electron Microscopy

Scanning electron microscopy (SEM) is a powerful technique frequently used to examine melanin and its particle size distribution. This pigment usually appears in the form of granules that have an amorphous morphology and an irregular shape, which can differ based on different structures and the source of origin [36]. The granules are generally made by layers with a pore size of 1–4 nm in diameter [60]. This technique was applied to analyze, for example, the melanin produced by the marine *Streptomyces* sp. strain, which showed black particles with a smooth and irregular surface and a porous structure [43]. The purified melanin produced by *Streptomyces hyderabadensis* 7VPT5-5R showed an amorphous, irregular shape [37], while the purified melanin produced by *Streptomyces glaucescens* NEAE-H also showed a structure made of distinct small spheres [22]. Sometimes, SEM helped in identifying changes in the appearance of melanin before and after the purification procedure. For example, analyses conducted on the melanin precipitated by the broth supernatant of *Streptomyces cavourensis* SV 21 exhibited a fluffy and cloudy appearance, while, after a further purification, the pigment displayed a consistently amorphous shape, i.e., a conglomerate of diverse irregular structures, possibly originating from its intricate branching and filamentous arrangement [46].

## 7. Properties of Melanin Produced by Streptomyces Strains

### 7.1. Physicochemical Properties

Pigments produced by different *Streptomyces* strains show many of the physicochemical characteristics of the melanin of animal origin. Melanins produced by *Streptomyces* strains were demonstrated to be soluble in aqueous alkali (i.e., 0.1 M NaOH) and DMSO but insoluble in acid solutions with a pH lower than 2.0. Some of these melanins were completely or slowly soluble in water, as in the case of the pigments produced by *S. roseochromogenes* ATCC 13400 and *S. nashvillensis* DSM 40314 [23,47], while in the case of *Streptomyces* sp. ZL-24 [32], *Streptomyces lusitanus* DMZ-3 [52], *Streptomyces* sp. [43], and *Streptomyces cavourensis* SV 21 [46], a contemporary production of both soluble and insoluble forms of melanin was observed. In addition, the pigments by *S. lusitanus* DMZ-3 [52], *S. roseochromogenes* ATCC 13400 [23], and *S. nashvillensis* DSM 40314 [47] were not soluble in alcohols or organic solvents like acetone or ethyl acetate, in contrast to the pigment produced by *Streptomyces kathirae* SC-1 [31]. In many papers, not only solubility but also melanin stability was tested. The melanin produced by *S. kathirae* SC-1 [31], *S. roseochromogenes* ATCC 13400 [23], and *S. nashvillensis* DSM 40314 [47] was found to be very stable in a wide range of temperatures between 40 and 100 °C (up to 95% of stability at 100 °C) and also highly resistant to the UV-VIS light exposure (up to 100% stability after 6 h of exposition). *Streptomyces* melanins were also demonstrated to be able to chelate ions of the transition metals like Fe (II or III), Cu (II), Mn (II), and Co (II), as reported for the pigments produced by *S. kathirae* SC-1 [31], *S. cavourensis* RD8 [40], and *S. roseochromogenes* ATCC 13400 [23], or metal ions like Ni (II) and Zn (II), as reported for the melanin produced by *S. nashvillensis* DSM 40314 [47]. Instead, they did not chelate alkaline and alkali earth metal ions like Na (I), K (I), Ca (II), or Mg (II). Melanin exhibited very low oxidability and reducibility properties (lower than 9.0% in 48 h), as in the case of pigments produced by *S. kathirae* SC-1 (15), *S. roseochromogenes* ATCC 13400 [23], and *S. nashvillensis* DSM 40314 [47], while they showed high antioxidant activity, in a concentration-dependent manner, in the range of 61.2–96.2% for the melanin produced by *Streptomyces kathirae* SC-1 [31], 75.0–85.0% for the pigment produced by *Streptomyces cavourensis* RD8 [40], up to 70.2% for the melanin by *Streptomyces* sp. [43], 70.7–92.2% for the melanin produced by *S. roseochromogenes* ATCC 13400 [23], and 93.5–96.9% for the pigment produced by *S. nashvillensis* DSM 40314 [47].

### 7.2. Biological Activities

**Figure 6:**
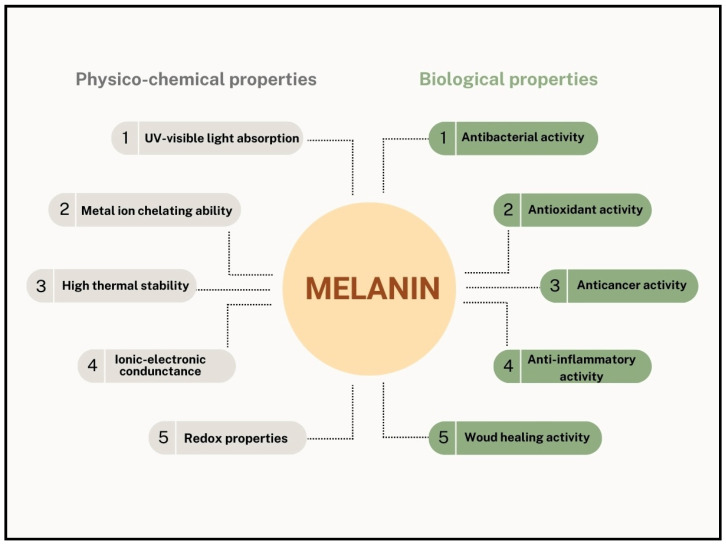
Physico-chemical and biological properties of the melanin pigments produced by *Streptomyces* strains, as tested, identified, and reported in the literature.

The melanin pigments produced by Streptomycetes were demonstrated to have numerous biological activities (Figure 6). They frequently showed antimicrobial activities in a concentration-dependent manner against numerous *Gram-positive* and *Gram-negative* bacteria, but not anti-fungal activity. Several papers tested the produced melanin at different concentrations against a wide range of different microorganisms by using agar well diffusion assay. For example, the brown pigment produced by *S.* BJZ10 was demonstrated to have an antibacterial activity against *Escherichia coli* (inhibition size diameter around 0.4 cm) and *Bacillus cereus* (inhibition size diameter around 1.4 cm) when used at 50 mg/mL [28], while *Streptomyces puniceus* RHPR9 melanin demonstrated inhibitory effects against *B. cereus* (inhibition size diameter around 2.5 cm) and *K. pneumoniae* (inhibition size diameter around 1.2 cm) when used at the concentration of 0.25 mg/mL [36]. Melanin showed anti-microbial activity even if it was not purified, as in the case of the crude pigment produced by *Streptomyces bellus* MSA1 that demonstrated high activity against *Escherichia coli* and *Staphylococcus aureus*, as well as against *Bacillus* sp. and *Pseudomonas aeruginosa* (inhibition size diameters between 0.7 and 0.8 cm) [29]. Despite this, purified melanin might show higher effectiveness, as in the case of the purified pigment from *S. cavourensis* SV 21 that showed around 80% anti-bacterial activity against the *Gram-positive* strain *Rhodococcus corynebacterioides* when used at 0.1 mg/mL concentration, while the crude precipitated one showed around 60% [46]. The purified melanin from *S. cavourensis* SV 21 was demonstrated to have no inhibitory effect but had anti-quorum sensing activity against the marine bacterium *Alivibrio fischeri* at a concentration of 100 μg/mL. This might suggest a potential role of melanin in defense mechanisms against other competing bacteria and a possible use in biotechnological systems to ecologically control microbe–host and/or microbe–microbe interactions [46]. Sometimes, the antimicrobial activity of the pigment also depends on its solubility. In fact, the water-soluble form of the melanin produced by *Streptomyces* sp. ZL-24 demonstrated potent antibacterial properties against the *Gram-positive S. aureus* ATCC 6538 and *M. smegmatis* ATCC 10231, as well as against the *Gram*-*negative* bacteria *P. aeruginosa* ATCC 9027 and *E. coli* ATCC 8379, when used in concentrations between 50 and 150 mg/mL (inhibition size diameters between 1.5 and3.6 cm) [32]. In the case of *M. smegmatis* ATCC 10231 and *P. aeruginosa* ATCC 9027, the melanin-inhibiting effect was due to its capacity to reduce the biofilm formation process in a dose-dependent manner. Instead, the melanin insoluble form just hindered the growth of the *Gram-positive* strains. However, both melanin pigments did not exhibit antifungal activities against *C. albicans* ATCC 10231 or *F. oxysporum* MTCC 387 [32]. Melanin produced by *Streptomyces* strains showed the ability to counteract very different microorganisms, as in the case of the melanin purified from the marine *Streptomyces* sp. MVCS13 that had antibacterial effects against ornamental fish bacterial pathogens like *Vibrio* sp. FPO5 (inhibition size diameter around 1.5 cm) and *Aeromonas* sp. FPO6 (inhibition size diameter around 1.2 cm), both isolated from *Carassius auratus*-infected fishes [44]. *Streptomyces* melanin also showed a slightly lower effect than the one of a standard streptomycin antibiotic even at a minimum inhibitory concentration (MIC) of 0.018 mg/mL [44]. *Streptomyces* melanins are also well known for their antioxidant properties. They were frequently tested at different concentrations, in diverse conditions, sometimes after a purification process, for their 2,2-diphenyl-1-picrylhydrazyl (DPPH), hydrogen peroxide (H_2_O_2_), 2,2′-azino-bis (3-ethylbenzothiazoline-6-sulfonic acid) (ABTS), or hydroxyl radical scavenging ability. For example, the antioxidant activity of the purified melanin produced by *Streptomyces cavourensis* RD8 was assayed at different pH values (7.5–12.5), temperatures (from 30 to 70 °C), and diverse sodium chloride (NaCl) concentrations (between 5% and 20% *w*/*v*) for both its DPPH and H_2_O_2_ scavenging ability [40]. At pH 8.0, the pigment demonstrated about 28% and 87% antioxidant DPPH and H_2_O_2_ activities, respectively; at 50 °C, the activities were 62% and 84%, respectively, while values around 55% and 75%, were also observed at a 5% NaCl concentration [40]. Both the crude and the purified melanin produced by *Streptomyces bellus* MSA1 showed around 82% DPPH scavenging activity [29]. The eumelanin produced by *Streptomyces parvus* BSB49 was tested at a concentration of 0.250 mg/mL for its ability to scavenge both DPPH and ABTS radicals, exhibiting an activity of about 88% and 75%, respectively [30]. Both the purified soluble and insoluble melanin produced by *Streptomyces* sp. showed significant free radical scavenging capabilities in a concentration-dependent manner. The insoluble melanin showed around 96% hydroxyl radical, 65% DPPH, and 60% H_2_O_2_ scavenging activity at a concentration of 50 μg/mL [32]. At the same concentration, the soluble melanin showed, instead, about 87% hydroxyl radical, 56% DPPH, and 61% H_2_O_2_ scavenging activity [32]. Melanin precipitated from *S. cavourensis* SV 21 supernatant exhibited about 80% (DPPH) scavenging at a 0.16 mg/mL concentration [46], while the purified eumelanin pigment from the *Streptomyces glaucescens* NEAE-H showed ABTS antioxidant properties of about 57% at a concentration of 0.100 mg/mL [22]. At the same concentration, the melanin produced by *Streptomyces puniceus* RHPR9 exhibited a DPPH antioxidant activity of about 89% [36]. This melanin also showed anticancer activity against HEK 293, HeLa, and SK-MEL-28 cell lines with IC50 values of 64.11, 14.43, and 13.3 μg/mL, respectively, according to the in vitro MTT assay [30]. In addition, this pigment also showed a wound-healing effect when tested on HeLa cells [36]. The melanin produced by *Streptomyces glaucescens* NEAE-H showed anticancer activity against the skin cancer cell line HFB4 with an IC50 value of 16.34 μg/mL [22]. Both of the melanin pigments produced by *Streptomyces puniceus* RHPR9 and *Streptomyces glaucescens* NEAE-H, instead, showed anti-haemolytic activity in vitro when tested on erythrocytes thanks to the ability of the phenolic groups to neutralize free radicals and thus to protect erythrocytes membranes from destruction and lysis (minimum hemolysis values were around 21.4 and 11.9%, respectively) [22,36]. Furthermore, the protective in vitro anti-inflammatory effect of the melanin produced by *Streptomyces puniceus* RHPR9, at a dose of 500 μg/mL, was comparable to that of commercial drugs (i.e., Diclofenac) at similar concentrations (78.6 and 80.7%, respectively) [36].

## 8. Applications of Melanin Produced by *Streptomyces* Strains

In a few cases, the melanin pigments produced by *Streptomyces* strains were also employed in some specific applications as dyes. For example, the melanin produced by *Streptomyces bellus* MSA1 was used as a pigment to impart the color of a bio-lip balm manufactured by using lanolin, coconut oil, and shredded bee wax [29]. Also, the melanin produced by *S. glaucescens* was employed to dye cotton and functional fibers; furthermore, by adding copper ions during the process, the dyeing speed increased by 86.9% [45].

## 9. Conclusions

The global market size of melanin in 2022 has been estimated at around USD 13.7 million, and it was calculated that it will reach USD 18 million by 2028, with a growth rate of 4.5%. From this perspective, microbial melanin might be an eco-friendly, sustainable, and valid alternative to animal-extracted pigments. Compared with other microorganisms, like fungi, *Streptomyces* strains can produce melanin in shorter times and as an extracellular product, which is easier to purify. As the melanin production by *Streptomyces* greatly depends on the strain and growth conditions, in the literature, many papers reported the isolation and the informed selection of new strains, as well as the methods to optimize the growth conditions and the medium composition to boost melanin production. The scaling up of the production in fermentation processes has been rarely investigated so far, and very few molecular biology strategies have been applied to obtain producer recombinant strains. Different purification processes, always at the lab scale, have been set up so far, while complete melanin structural characterizations have been widely performed by using multi-analytical approaches. The wide range of physicochemical and biological properties of the melanins produced by *Streptomyces* strains, similar to those of animal-origin pigments, might open many new possibilities for use in different industrial sectors, like in pharmaceutical and biomedical fields and in food packaging and dyeing industries, as well as in environmental bioremediation and in bioelectronic and biosensors.

## Figures and Tables

**Table 1:** Growth parameters, medium composition, and fermentation conditions used to produce melanin by different *Streptomyces* strains, as reported in the literature.

Streptomyces Strain	Melanin Production (g/L)	Medium Components (g/L)	Production System	Growth Conditions	Ref.
*S*. BJZ10	3.0	Soluble starch (10.0), casein (0.30), KNO_3_ (2.0), MgSO_4_·7 H_2_O (0.05), K_2_HPO_4_ (2.0), NaCl (2.0), CaCO_3_ (0.02), FeSO_4_·7 H_2_O (0.01)	Shake flask	Room temperature, 120 rpm, 30 days	[28]
*S. cavourensis* RD8	0.087	Casein (1.0), L-tyrosine (0.15), NaNO_3_ (1.0), agar (2.5)	Agar plate	pH 7.0, 30 °C, 120 rpm, 7 days	[40]
*S*. *bellus* MSA1	not reported	Dextrose (0.1), casein (0.1), MgSO_4_·7 H_2_O (0.5), FeSO_4_·7 H_2_O (0.01), NaCl (0.5), K_2_HPO_4_ (2.28)	Shake flask	pH 6.0, 8.0, 35–40 °C, 10 days	[29]
*S. parvus* BSB49	0.160–0.240	Dextrose (4.0), malt extract (10.0), yeast extract (4.0), agar (20.0) Soluble starch (10.0), MgSO_4_·7 H_2_O (1.0), NaCl (1.0), (NH_4_)_2_SO_4_ (2.0), CaCO_3_ (2.0), 1 mL of trace salt solution [FeSO_4_·7 H_2_O (0.01), MnCl·4 H_2_O (0.01) ZnSO4·7 H_2_O (0.01)], agar (20.0)	Agar plate	30 °C, 4–5 days	[30]
*S*. *glaucescens* NEAE-H	0.35	Peptone (15.0), proteose peptone (5.0), yeast extract (1.0), ferric ammonium citrate (0.5), K_2_HPO_4_ (1.0), Na_2_S_2_O_3_ (0.08)	Shake flask	30–37 °C, 100–200 rpm, 3–6 days	[22]
*S. kathirae*	13.7	Amylodextrine (3.3), yeast extract (37.0), NaCl (5.0), CaCl_2_ (0.1), CuSO_4_ (0.69), L-tyrosine (0.00025)	Shake flask	pH 6.0, 28 °C, 200 rpm, 2 days	[31]
*S.* DMZ-3	0.264	Gelatin (5.0), L-tyrosine (5.0), beef extract (3.0), agar (20.0)	Agar plate	pH 8.5, 50 °C, 5 days	[52]
*S. roseochromogenes* ATCC 13400	3.94 (shake flask), 9.20 (batch fermentation)	Glucose (12.0), yeast extract (6.0), malt extract (30.0), KH_2_PO_4_ (42.9), K_2_HPO_4_ (17.4), egagropili powder (2.5)	Shake flask, batch fermentation (1.8 L)	pH 6.0, 26 °C, 250 rpm, 5 days (shake flasks), 4 days (batch)	[23]
*S.* ZL-24	0.189 (insoluble melanin), 4.24 (soluble melanin)	Soy peptone (20.3), NiCl_2_ (0.39), FeSO_4_ (1.33)	Shake flask	pH 7.0, 30 °C, 5 days	[32]
*S. antibioticus* *NRRL B-1701*	0.244	Soluble starch (4.92), yeast extract (5.16), CuSO_4_ (0.019), L-tyrosine from *Arthrospira platensis* (Spirulina), hydrolysate (17.11), NaCl (5.0), CaCl_2_ (0.1)	Shake flask	pH 6.0, 30 °C, 150 rpm, 7 days	[39]
*S*. MVCS 13	0.147	Glycerol (10.0), L-tyrosine (0.5), L-asparagine (1.0), K_2_HPO_4_ (0.5), MgSO_4·_7 H_2_O (0.5), NaCl (0.5), FeSO_4·_7 H_2_O (0.01), trace salt solution	Shake flask	pH 7.0, 28 °C, 200 rpm, 7 days	[44]
*S. cavourensis* SV 21	0.116	Peptone (5.0), yeast extract (1.0), C_6_H_5_FeO_7_ (0.1), NaCl (19.45) MgCl_2_ (5.9), MgSO4 (3.24), CaCl_2_ (1.8), KCl (0.55), NaHCO_3_ (0.16), KBr (0.08), SrCl_2_ (0.034), H_3_BO_3_ (0.022), Na_2_SiO_3_ (0.004), NaF (0.0024), NH_4_NO_3_ (0.0016), Na_2_HPO_4_ (0.008)	Shake flask	30 °C, 14 days	[46]
*S. cyaneus*	9.9 (with 2.0% fava bean seed peel supplementation) 11.1 (with gamma irradiation)	Soluble starch (20.0), NaNO_3_ (0.5), MgSO_4_·7 H_2_O (0.5), KCl (0.5), K_2_HPO_4_ (1.0), CaCO_3_ (2.0)	Shake flask	37 °C, 200 rpm, 7 days	[33]
*S.* sp.-EF1	0.082	Starch (50), NaNO_3_ (2.0), K_2_HPO_4_ (1.0), MgSO_4_·7 H_2_O (0.5), KCl (0.5), FeSO_4_·7 H_2_O (0.01)	Shake flask	pH 7.2, 22 °C, 120 rpm	[56]
*S. puniceus* RHPR9	0.386	Peptone (15.0), proteose peptone (5.0), yeast extract (1.0), ferric ammonium citrate (0.5), K_2_HPO_4_ (1.0), Na_2_S_2_O_3_ (0.08)	Shake flask	pH 6.7, 30 °C, 150 rpm, 7 days	[36]
*S.* 7VPTS-SR	5.54	Peptone (15.0), proteose peptone (5.0), yeast extract (1.0), ferric ammonium citrate (0.5), K_2_HPO_4_ (1.0), Na_2_S_2_O_3_ (0.08)	Shake flask	pH 6.7, 28 °C, 7–10 days	[37]
*S. nashvillensis*	0.74	Glucose (12.0), yeast extract (6.0), malt extract (30.0), NaH_2_PO_4_·H_2_O (5.8), Na_2_HPO_4_ (8.2)	Shake flask	pH 7.0, 28 °C, 250 rpm	[47]
*S. djakartensis* NSS-3	11.8	Peptone (15.0), proteose peptone (5.0), yeast extract (1.0), ferric ammonium citrate (0.5), K_2_HPO_4_ (1.0), Na_2_S_2_O_3_ (0.08), L-tyrosine (2.0)	Shake flask	30 °C, 160 rpm, 7 days	[35]
*S*. MR28	0.6	Glycerol (15.0), L-tyrosine (0.5), L-asparagine (1.0), MgSO_4_·7 H_2_O (0.5), K_2_HPO_4_ (0.5), NaCl (0.5), with 1 mL of trace salt solution (FeSO_4_.7 H_2_O (0.00136), CuCl_2_·2 H_2_O (0.000027), CoCl_2_·6 H_2_O (0.00004), Na_2_MoO_4_·6 H_2_O (0.000025), H_3_BO_3_ (0.0028), ZnCl_2_ (0.00029), C_4_H_4_Na_2_O_6_ (0.0018), MnCl_2_·4 H_2_O (0.0018)	Shake flask	pH 6.0–8.0, 20–40 °C, 10 days	[34]

**Table 2:** Summary of the different protocols, reported in the literature, for melanin purification.

Streptomyces Strain	Isolation and Purification	Ref.
*S.* BJZ10	An equal volume of different solvents including ethanol, methanol, acetone, propanol, hexane, water, and a supernatant sample was taken and mixed well. The mixture was centrifuged at 8000 rpm for 10 min. The supernatant was monitored at 540 nm to check the optical density.	[28]
*S. cavourensis* RD8	The supernatant was neutralized by the addition of 1.0% potassium per-sulphate. The mixture was allowed to stand for 2 h at room temperature with intermittent shaking. After 2 h, half the volume of methanol was added to the total solution and kept for 72 h at room temperature. The solution was centrifuged, the pellets were dissolved in alkaline distilled water, and the pH was adjusted to 3.0. The precipitates were washed with alkaline distilled water and dialyzed followed by lyophilization. Melanin purification was performed using ion exchange chromatography using a Sephadex LH-20 column, pre-equilibrated with 20 mM potassium phosphate buffer (pH 7.0). The bound melanin was eluted by buffer at a flow rate of 20 mL/h, and fractions (2 mL) were assayed for the presence of melanin.	[40]
*S. roseochromogenes*	Supernatants were precipitated by the addition of glacial acetic acid up to pH 1.0. The sample was kept at 4 °C for 18 h and then centrifuged at 4 °C and 4500 rpm for 20 min. The collected precipitated material was washed three times with MilliQ water. The material was centrifuged at 4 °C and 4500 rpm for 20 min. The obtained material was solubilized in 0.1 M NaOH at a concentration of 1.5 g∙L^−1^ and purified by using a preparative chromatographic system.	[23]
*S. parvus* BSB49 *S. glaucescens* NEAE-H *S. kathirae* *S.* ZL-24 *S. glaucescens* *S. puniceus* RHPR9	The pH of the supernatant was adjusted to 2.0 by 6 M HCl. The solutions were stored at room temperature or 4°C for 3–4 h or 24 h or 48 h. The solutions were centrifuged at 5000–10,000 rpm for 10–20 min. The pellets were washed with distilled water 3–4 times. The pellets were centrifuged at 9000–10,000 rpm for 10–15 min.	[22,30,31,32,36,45]
*S. lusitanus* DMZ-3	Intracellular melanin: Cells were suspended in 0.05 M Na_2_HPO_4_ pH 7.5 and sonicated. The extracted solution was hydrolyzed several times for 20 days with concentrated HCl. Washing was performed several times using 5% (*w*/*v*) HCl. The solution was extracted with ethanol THF for 20 h. The extracted dried pigment pellet was subjected to dialysis in a cellulose membrane against phosphate buffer at pH 7.0. Purification was performed by column using silica gel material of a 60–120 mesh size. Extracellular melanin: The pigment was recovered by acidifying the supernatant and extracting with butanol. The extracted dried pigment pellet was subjected to dialysis in a cellulose membrane against phosphate buffer at pH 7.0. Purification was performed by column using silica gel material of a 60–120 mesh size.	[52]

**Table 3:** Maximum wavelengths of the UV absorbance of the melanin pigments produced by *Streptomyces* strains, as reported in the literature.

Strain	Maximum UV Wavelength (nm)	Reference
*S*. BJZ10	247	[28]
*S. parvus* BSB49	250	[30]
*S. glaucescens* NEAE-H	250	[22]
*S. puniceus* RHPR9	250	[36]
*S*. ZL-24	Insoluble: 207, soluble: 213	[32]
*S. lusitanus* DMZ-3	Insoluble and soluble 230	[52]
*S. kathirae* SC-1	220	[31]
*S. roseochromogenes ATTC*	222, 254	[23]
*S*. MVCS13	300	[44]
*S. cavourensis* SV 21	260	[46]
*S. nashvillensis DSM 40314*	220	[47]

**Table 4:** FT-IR signals of the melanin produced by different *Streptomyces* strains, as reported in the literature.

Strain	FT/IR Signals (cm^−1^)															
	O-H N-H	Aliphatic C-H	Amine, Amide, Carboxylic Acid	C=O	Aromatic C=C	N-H	CH_2_-CH_3_	S=O	Aromatic Esters	C-OH	Aliphatic C-N	Aliphatic C-H	Aromatic C-H	C-S	Type of Melanin	Ref.
*Streptomyces* BJZ10	3362	2902						1388	1310–1250						n.d.	[28]
*Streptomyces cavourensis* RD8	3346	2943				1654					1112, 1029				Pheomelanin	[40]
*Streptomyces bellus* MSA1	3346	2943				1654					1112, 1029				n.d.	[29]
*Streptomyces parvus* BSB49	3265	2923	2500			1632, 1528	1450			1210					Eumelanin	[30]
*Streptomyces* sp.	3362				1645	1645, 1532	1451							662	Pheomelanin	[43]
*Streptomyces glaucescens* NEAE-H	3421	2947	2800		1647	1647, 1539	1423		1305–1243	1240		1058	864		Eumelanin	[22]
*Streptomyces kathirae* SC-1	3200–3400	2919			1631										n.d.	[31]
*Streptomyces lusitanus* DMZ-3	3305 (insoluble), 3386 (soluble)				1651 (insoluble), 1644 (soluble)										n.d.	[52]
*Streptomyces* sp. ZL-24	3375 (insoluble), 3282 (soluble)	2962 (insoluble), 2929 (soluble)			1631 (insoluble), 1627 (soluble)	1515 (insoluble and soluble)	1452 (insoluble), 1450 (soluble)			1259 (insoluble and soluble)		1095 (insoluble), 1078 (soluble)	802 (insoluble and soluble)		Eumelanin	[32]
*Streptomyces* F1, F2, F3	3381	2925			1645	1633									Eumelanin	[42]
*Streptomyces* sp. MVCS13	3422	2959, 2924	2343, 2361		1625						1096, 1035				n.d.	[44]
*Streptomyces glaucescens*	3420	2920, 2850		1713	1623										n.d.	[45]
*Streptomyces parvus* BSB49	3265	2923	2500			1632, 1528	1450			1210					Eumelanin	[30]
*Streptomyces puniceus* RHPR9	3443, 3421	2956	2800		1650	1629				1246		1051	835		Eumelanin	[36]
*Streptomyces nashvillensis* DSM 40314	3464	2920, 2851	2363, 2338	1725	1647	1647	1405			1233-1153		1075	872		Eumelanin	[37]
